# Functional cooperation between KCa3.1 and TRPC1 channels in human breast cancer: Role in cell proliferation and patient prognosis

**DOI:** 10.18632/oncotarget.9261

**Published:** 2016-05-10

**Authors:** Malika Faouzi, Frederic Hague, Dirk Geerts, Anne-Sophie Ay, Marie Potier-Cartereau, Ahmed Ahidouch, Halima Ouadid-Ahidouch

**Affiliations:** ^1^ University of Picardie Jules Verne, UFR of Sciences, EA4667 Laboratory of Cell and Molecular Physiology, SFR CAP-SANTE (FED 4231), Amiens, France; ^2^ Queen's Center for Biomedical Research, The Queen's Medical Center, Honolulu, HI 96813, USA; ^3^ Department of Pediatric Oncology/Hematology, Erasmus University Medical Center, 3015 GE Rotterdam, The Netherlands; ^4^ Inserm, UMR1069, Nutrition, Growth and Cancer, University of François Rabelais, Tours F-37032, France

**Keywords:** breast cancer, cell proliferation, calcium, KCa3.1, TRPC1

## Abstract

Intracellular Ca2^+^ levels are important regulators of cell cycle and proliferation. We, and others, have previously reported the role of KCa3.1 (KCNN4) channels in regulating the membrane potential and the Ca2^+^ entry in association with cell proliferation. However, the relevance of KC3.1 channels in cancer prognosis as well as the molecular mechanism of Ca2^+^ entry triggered by their activation remain undetermined. Here, we show that RNAi-mediated knockdown of KCa3.1 and/or TRPC1 leads to a significant decrease in cell proliferation due to cell cycle arrest in the G1 phase. These results are consistent with the observed upregulation of both channels in synchronized cells at the end of G1 phase. Additionally, knockdown of TRPC1 suppressed the Ca2^+^ entry induced by 1-EBIO-mediated KCa3.1 activation, suggesting a functional cooperation between TRPC1 and KCa3.1 in the regulation of Ca2^+^ entry, possibly within lipid raft microdomains where these two channels seem to co-localize. We also show significant correlations between KCa3.1 mRNA expression and poor patient prognosis and unfavorable clinical breast cancer parameters by mining large datasets in the public domain. Together, these results highlight the importance of KCa3.1 in regulating the proliferative mechanisms in breast cancer cells as well as in providing a promising novel target in prognosis and therapy.

## INTRODUCTION

Breast cancer is one of the most frequent malignancies in women worldwide. There have been striking advances in breast cancer therapy over the last decade. However, patients with advanced disease typically respond poorly and many still die of the complexity of refractory breast cancers. Understanding the regulatory mechanisms that influence cancer cell growth and survival is the key to developing new targeted therapies and more efficacious pharmacological approaches. Ion channels of the K^+^ and TRP (Transient Receptor Potential) channel families are very important players in the regulation of specific stages of cancer progression, and are increasingly studied as promising targets for novel molecular cancer therapies [1–10].

Among the K^+^ channels, the intermediate-conductance Ca^2+^-activated K^+^ channels (KCa3.1, gene name KCNN4) have been widely studied in many cancers. Indeed, Jäger and collaborators have reported 6-66 fold increase of KCa3.1 mRNA levels in 89% of the tested primary pancreatic tumor tissues as compared to non-tumor tissues [11]. Similar results were found in prostate cancer [12] as well as in endometrial cancer, where KCa3.1 showed expression levels that were higher in tumor than in normal tissues, but that were also higher in cancer than in atypical hyperplasia [13]. KCa3.1 expression level has also been correlated with breast tumor grade. These findings suggest that KCa3.1 channels are important for cancer cell proliferation, and that their blockade might prove useful as therapy in patients with up-regulated KCa3.1 tumor expression. In this context, we have previously investigated the role and functional expression of KCa3.1 channels in controlling breast cancer cell cycle progression [14]. We have shown that KCa3.1 channels are involved in cell proliferation by regulating G1/S transition. Both KCa3.1 mRNA expression and channel activity increased during G1 phase progression, concomitant with increased Ca^2+^ entry. Nevertheless, the molecular mechanisms involved in this Ca^2+^ entry remain unknown.

Over the past few years, many studies have investigated the expression of Ca^2+^ channels in breast cancer cell lines in order to elucidate their involvement in regulating Ca^2+^ entry. Some of these studies have reported that TRPC1, TRPC6 and TRPM7, members of TRP channel family, are involved in cell proliferation as well as in regulating Ca^2+^ entry in breast cancer [15–18]. We also showed that TRPC1 mRNA and protein levels were higher in breast ductal adenocarcinoma tissues than in adjacent non-tumor tissues [3]. Consistent with a causal role of TRPC1 in breast cancer, Mandavill *et al.*, showed that TRPC1 expression increased in infiltrating ductal carcinoma samples with microcalcifications when compared with age-matched controls without calcification or cancer [19].

The present study aims to investigate the mechanisms by which KCa3.1 regulates Ca^2+^ entry leading to breast cancer cell proliferation. We show, for the first time, a strong endogenous interaction between KCa3.1 and TRPC1 channels and a potential co-localization in lipid rafts. This co-localization allows TRPC1 channels to control the Ca^2+^ entry mediated by KCa3.1 activation, triggering signaling pathways that promote cell proliferation. In addition, this study presents the first analyses of KCa3.1 expression in patient prognosis and other important clinical correlations in breast cancer datasets in the public domain. These strongly suggest that KCa3.1 is involved in breast cancer proliferation, making it a promising target for the development of novel therapies.

## RESULTS

### Both TRPC1 and KCa3.1 are involved in MCF-7 cell proliferation

**Figure 1:**
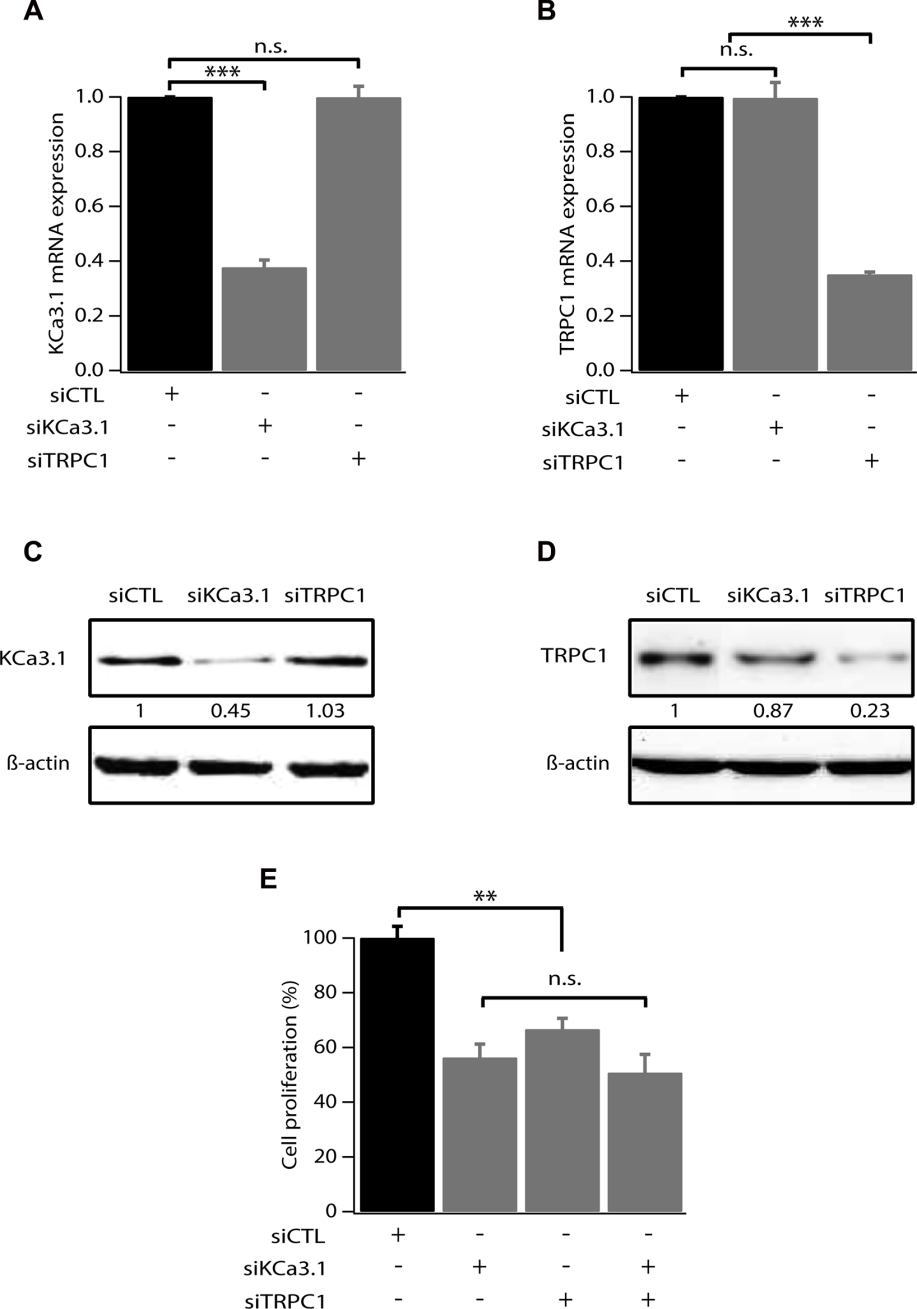
TRPC1 and KCa3.1 involvement in breast cancer cell proliferation (**A**) qRT-PCR analysis of KCa3.1 mRNA expression in MCF-7 cells transfected with scrambled siRNA (siCTL), siRNA directed against KCa3.1 (siKCa3.1), siRNA directed against TRPC1 (siTRPC1). The graph shows KCa3.1 mRNA expression normalized to β-actin mRNA expression (*n* = 4). (**B**) qRT-PCR analysis of TRPC1 expression level in MCF-7 cells transfected with siCTL, siKCa3.1 or siTRPC1. The graph shows TRPC1 mRNA expression normalized to b-actin mRNA expression (*n* = 4). (**C**) Representative western blot showing the effect of siRNAs directed against KCa3.1 and TRPC1 on the protein level of KCa3.1. (**D**) Representative western blot showing the effect of siRNAs directed against KCa3.1 and TRPC1 on the protein level of TRPC1. (**E**) Analysis of MCF-7 cell proliferation transfected with siCTL, siKCa3.1, siTRPC1 or both siKCa3.1 and siTRPC1. Cell proliferation is measured 72 h post-transfection. Values are reported as mean ± SEM normalized to the control (*n* = 4). **p < 0.01, ***p < 0.001, n.s.: not significant.

We have previously reported that hyperpolarization induced by KCa3.1 activation is a key element for cell cycle progression in MCF-7 breast cancer cells [14]. We have also demonstrated that MCF-7 cell proliferation can be dependent on Ca^2+^ entry through TRPC1 channels [15, 16]. Here we investigate whether KCa3.1 and TRPC1 use a common pathway in controlling cell growth. We first tested the efficacy of siRNA-KCa3.1 (siKCa3.1) and siRNA-TRPC1 (siTRPC1), used individually or in combination. As shown in Figure 1, 72 h treatment with siTRPC1 and siKCa3.1 significantly reduced the mRNA expression level of TRPC1 (64.9 ± 0.9% decrease, *p* = 7.37 · 10^−7^) and KCa3.1 (62.3 ± 2.6% decrease, *p* = 2.17 · 10^−5^), respectively (*n* = 4, Figure 1A, 1B). The knockdown efficacy was also significant at the protein level (55% decrease for KCa3.1 and 77% decrease for TRPC1). Additionally, TRPC1 silencing did not affect the level of KCa3.1 expression and neither did KCa3.1 silencing for TRPC1 expression level (Figure 1A–1D). Our results demonstrate that these two channels do not transcriptionally regulate each other. We then measured the effect of TRPC1 and KCa3.1 silencing on MCF-7 cell proliferation using a Trypan Blue assay. We found that the proliferation rate was significantly decreased in cells transfected with siTRPC1 (66.6 ± 4%; *p* = 0.005, *n* = 6) and siKCa3.1 (56.3 ± 5%; *p* = 0.003, *n* = 6) compared to siCTL (100 ± 4.2%). Interestingly, no additive or synergistic effects were observed in cells transfected with both siTRPC1 and siKCa3.1 compared to the effects obtained with siTRPC1 or siKCa3.1 (Figure 1E). Under all conditions, no significant cell mortality was detected.

**Figure 2:**
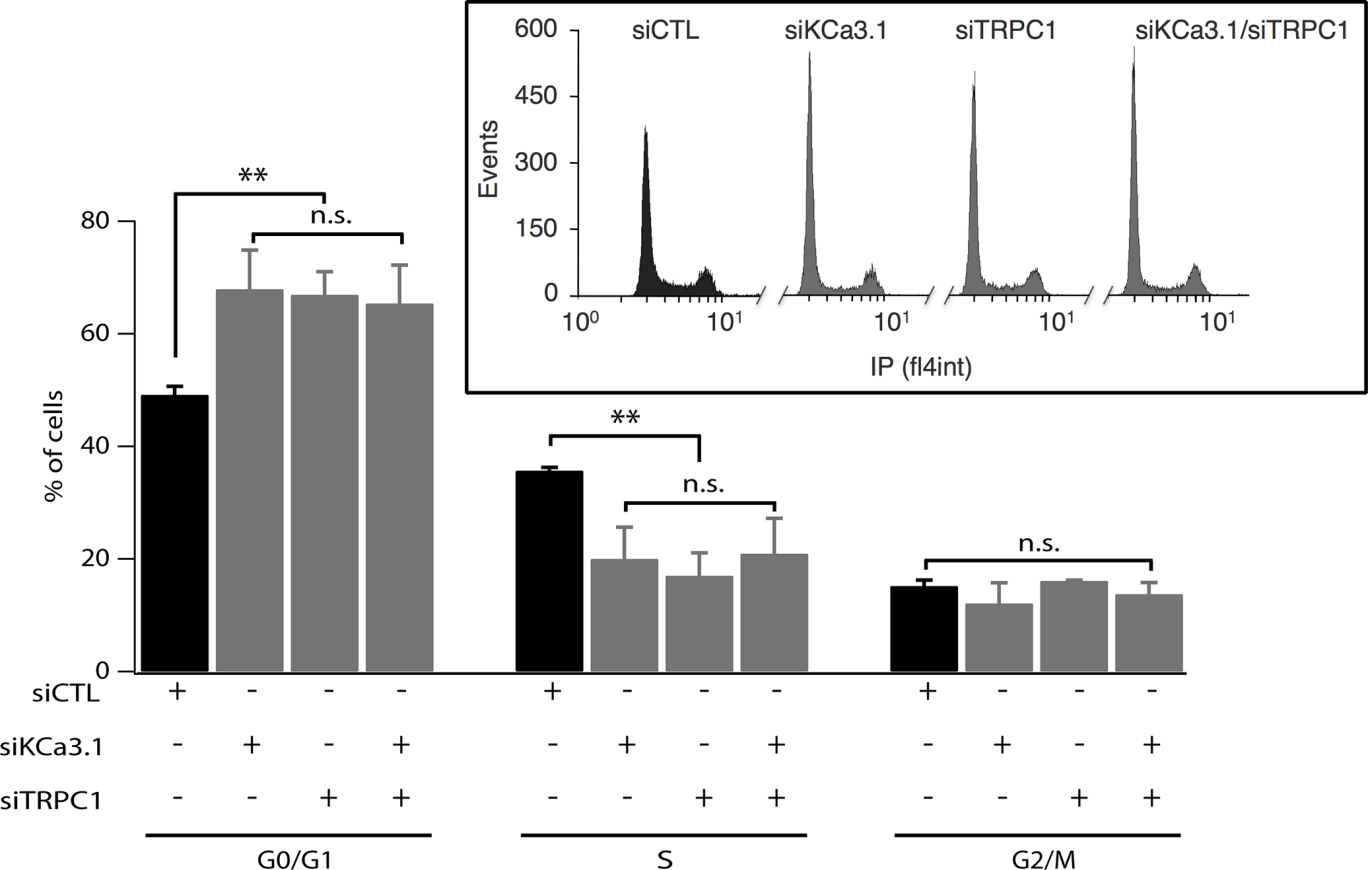
Silencing of TRPC1 and KCa3.1 expression induces accumulation of cells in G1 phase MCF-7 cells were transfected using Amaxa with either control siRNA (siCTL), KCa3.1 siRNA (siKCa3.1), TRPC1 siRNA (siTRPC1) or both TRPC1/KCa3.1 siRNAs (siTRPC1/siKCa3.1), and then cultured in EMEM medium with 5% FBS for 72 h. After staining with propidium iodide, cell cycle distribution (G0/G1, S and G2/M phases) was examined by flow cytometry. The graph represents the percentage of cells in different phases under control condition or KCa3.1 or TRPC1 knockdown conditions (*n* = 3). Insets show raw data from the FACS acquisition software. Values are reported as mean ± SEM. **, *p* < 0.01, n.s.: not significant.

To determine how siTRPC1 and siKCa3.1 affect cell proliferation, we performed cell cycle analysis using flow cytometry. Cell cycle distribution of MCF-7 ells transfected with siCTL was 49.17 ± 1.5%, 35.67 ± 0.6%, and 15.17 ± 1.06%, in G0/G1, S and G2/M phases, respectively (Figure 2). An accumulation in G0/G1 accompanied by a decrease in S phase was observed in cells transfected with siTRPC1 (66.93 ± 4.10%, 17 ± 4.05%, respectively, *n* = 3, *p* < 0.01). Similar results were obtained in MCF-7 transfected with siKCa3.1 (67.9 ± 6.94% in G0/G1 and 20 ± 5.65% in S, *n* = 3, *p* < 0.01). Again, no additive effect was observed in cells transfected with both siTRPC1 and siKCa3.1 in comparison with cells transfected with siTRPC1 or siKCa3.1 alone (Figure 2, *n* = 3). Taken together, our results suggest that TRPC1 and KCa3.1 regulate cell cycle progression in G1 phase and G1/S transition most likely through a common pathway.

### TRPC1 and KCa3.1 are over-expressed in end G1 phase

**Figure 3:**
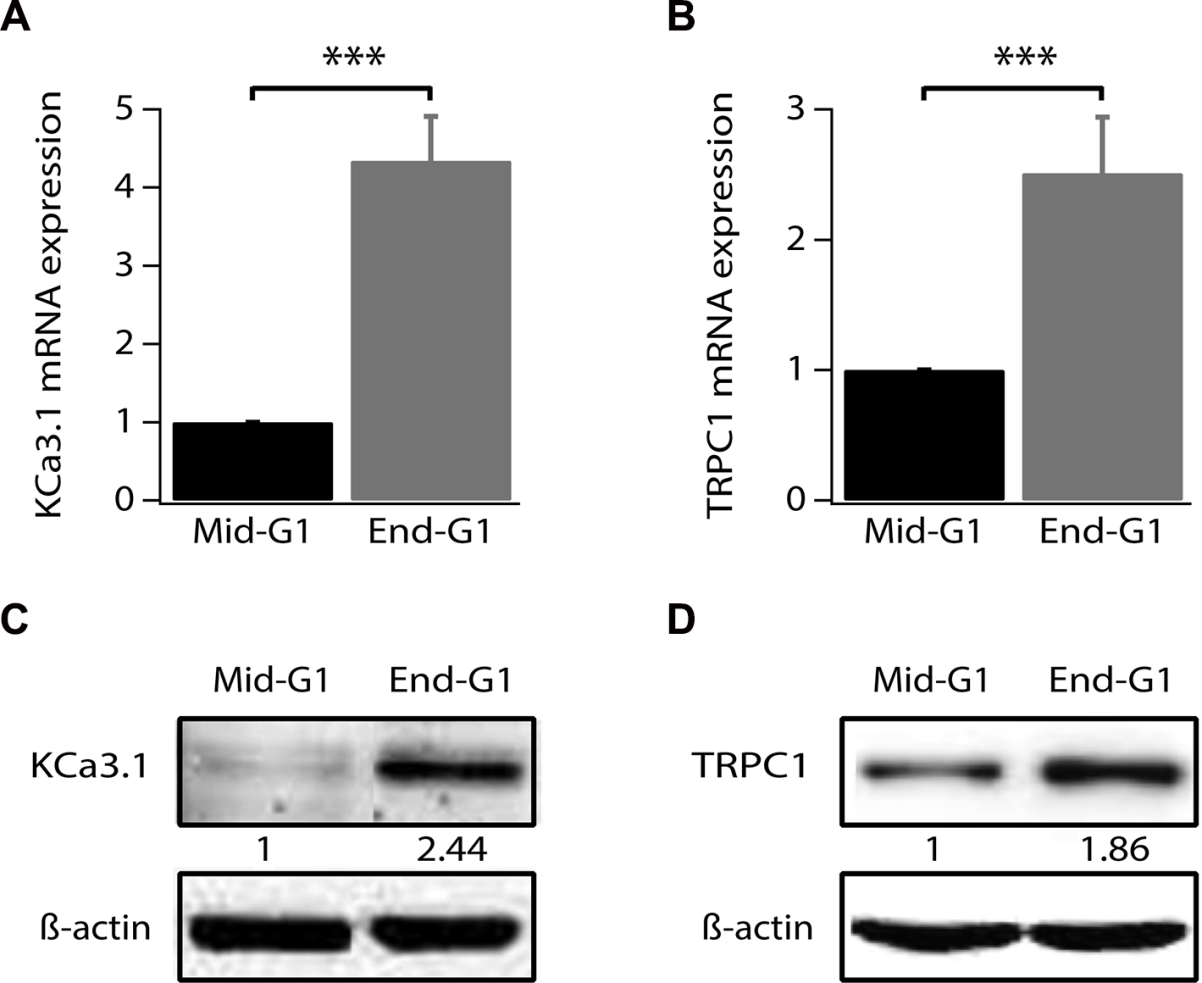
TRPC1 and KCa3.1 upregulation during G1 phase progression (**A**) qRT-PCR analysis of KCa3.1 mRNA expression level in synchronized cells. (**B**) qRT-PCR analysis of TRPC1 mRNA expression level in synchronized cells. (**C**) Representative western blot showing the expression level of KCa3.1 in synchronized cells. (**D**) Representative western blot showing the expression level of TRPC1 in synchronized cells. Cell synchronization was achieved by 24 h treatment of cells by either serum- and phenol red-free medium (mid-G1) or by complete medium supplemented with 2 mM thymidine (end-G1). Values are reported as mean ± SEM normalized to control. ***p < 0.001.

Our previous study has shown an increase of KCa3.1 mRNA in the end of G1 phase and during S phase [14]. However, changes in TRPC1 expression level during the cell cycle progression of breast cancer cells have never been reported. Given the fact that TRPC1 is also involved in MCF-7 cell proliferation and its knockdown induces accumulation of cells in G1 phase (Figures 1 and 2), we analyzed its expression in cells synchronized in early or late G1 (phase). Using quantitative reverse transcriptase PCR (qRT-PCR), we confirm that KCa3.1 mRNA level increases in end G1 phase compared to early G1 phase (Figure 3A, *p* < 0.001, *n* = 4). Additionally, we found that, like KCa3.1, TRPC1 expression increased in end G1 to reach 2.51-fold the level of expression in early G1 phase (Figure 3B, *p* < 0.001, *n* = 4). This upregulation was also reflected by an increase of protein expression (Figure 3C, 3D). Our results demonstrate that both TRPC1 and KCa3.1 are transcriptionally upregulated during the cell cycle progression supporting their role in the G1 phase and cell proliferation.

### KCa3.1 activation induces Ca^2+^ entry through TRPC1 channel

**Figure 4:**
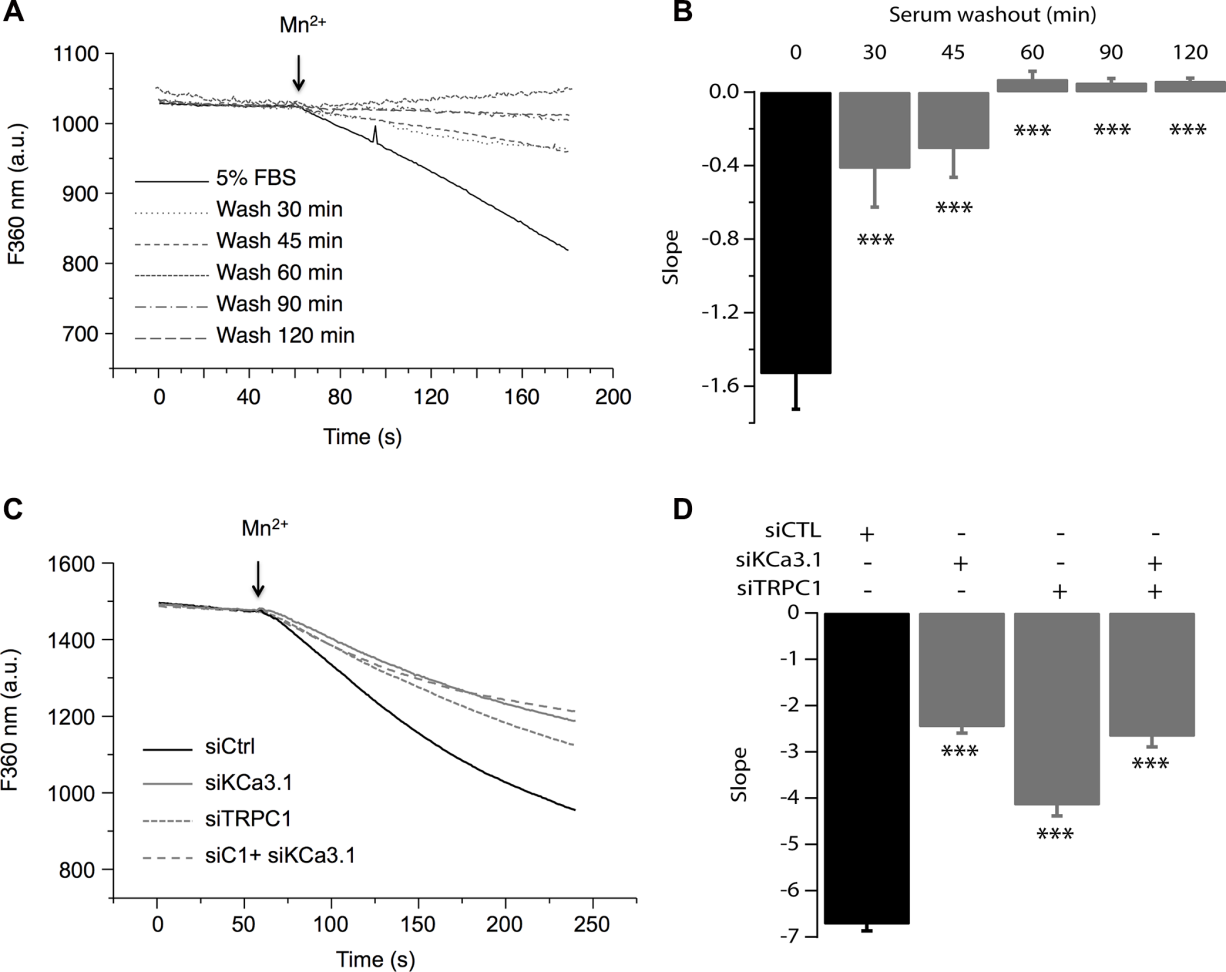
TRPC1 and KCa3.1 channels regulate the basal Ca^2+^ entry (**A**) Representative traces of Mn^2+^ quenching in cells at different time points after washing out the culture medium. (**B**) The graph represents the average slope value of 9 measurements for each condition. ***p < 0.001. (**C**) Representative traces of Mn^2+^ quenching in cells transfected with siCTL, siKCa3.1, or siTRPC1). Slope values are: −6.73 ± 0.14, for siCTL, −2.46 ± 0.13 for siKCa3.1, −4.15 ± 0.23 for siTRPC1 and −2.67 ± 0.22 for co-transfection of siKCa3.1 and siTRPC1. (**D**) The graph represents the average slope value of 31 measurements for each condition. ***p < 0.001.

Previous reports have demonstrated TRPC1 as a key player in both constitutive Ca^2+^ entry and Store Operated Calcium Entry (SOCE) [20–23] as well as in MCF-7 cell proliferation through ERK1/2 pathways [16]. We also have reported that KCa3.1 regulates Ca^2+^ influx by regulating the resting membrane potential (RMP) in the MCF-7 cell line [14]. Indeed, silencing KCa3.1 generates a strong depolarization of the RMP [24]. To further understand the relationship between TRPC1 and KCa3.1 in regulating BC cell proliferation, we investigated the involvement of TRPC1 in both basal and KCa3.1-regulated Ca^2+^ influx. The basal Ca^2+^ influx was measured indirectly using Mn^2+^ dye-quenching method where quenching of fura-2 fluorescence is induced by adding external manganese. In order to keep the channels active under the physiological conditions, cells were loaded with fura-2 in the presence of their growth medium. The culture medium was only washed out right before the calcium measurement. This step was critical to record the full basal activity, since removal of serum significantly decreased the quenching slope (Figure 4A, 4B). This time-dependent effect of the washout would be the result of ion channels deactivation due to the removal of all stimuli. As it is well established that serum contains growth factors that trigger store-operated calcium entry and calcium oscillations, hence activating any store-operated calcium channels and calcium-activated channels. After establishing the optimal recording conditions, we then measured the contribution of TRPC1 and KCa3.1 to the basal calcium influx. As illustrated in Figure 4 (Figure 4C, 4D) TRPC1 silencing decreased the dye-quenching slope by 38.25% *vs.* control (*p* = 7.47 · 10^−9^, Figure 4C, 4D). Using the same approach described above, we investigated the importance of the depolarization induced by KCa3.1 knockdown on the Ca^2+^ influx. Transfected cells with siKCa3.1 showed a stronger reduction of the quenching slope (thus Ca^2+^ influx) in comparison with the control (63.39%, *p* = 3.23 · 10^−16^, Figure 4C, 4D). The simultaneous knockdown of both TRPC1 and KCa3.1 resulted in a similar effect (60.31%, *p* = 3.13 · 10^−13^, Figure 4C, 4D). Taken together, our results showed that both TRPC1 and KCa3.1 regulate basal Ca^2+^ entry in MCF-7 cells. Additionally, combining the two siRNA did not result in an additive or synergistic effect, suggesting the presence of a functional cooperation between TRPC1 and KCa3.1.

**Figure 5:**
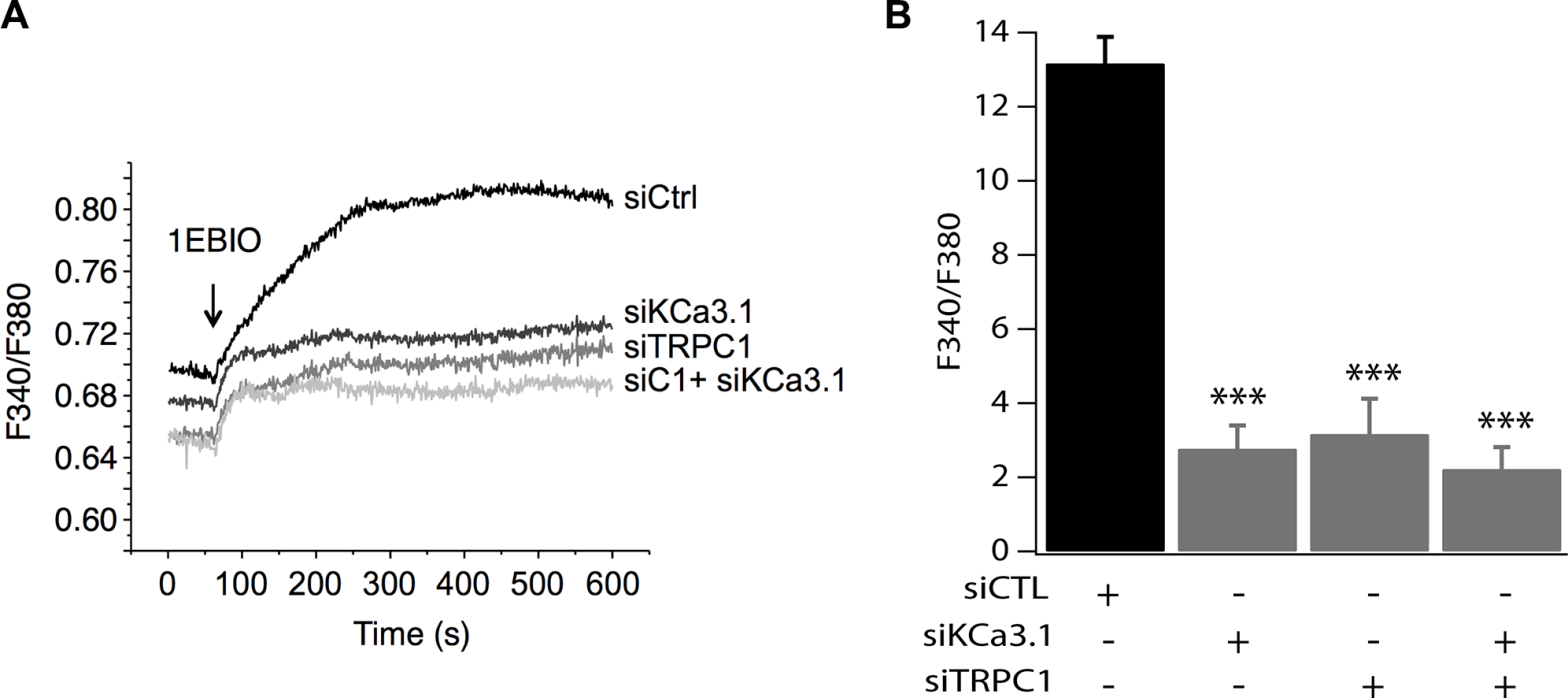
TRPC1 involvement in Ca^2+^ entry induced by KCa3.1 activation (**A**) Representative traces of 1-EBIO-induced Ca^2+^ entry. 1-EBIO was used at 200 μM. (**B**) Quantitative analysis of 1-EBIO-induced Ca^2+^ entry. The increase of [Ca^2+^]i induced by KCa3.1 activation is drastically reduced by siKCa3.1 and siTRPC1. ***p < 0.001.

To confirm our hypothesis, we studied the effect of pharmacological activation of KCa3.1 on the basal Ca^2+^ influx in cells transfected with siTRPC1 and/or siKCa3.1. As mentioned above, we know that KCa3.1 channels regulate the membrane potential. Thus, their activation will hyperpolarize the plasma membrane and consequently enhance the driving force for Ca^2+^. These effects are illustrated in Figure 5 where we can see that in the control cells, the extracellular perfusion of 200 μM 1-EBIO induced a significant increase in the basal Ca^2+^ influx while in siKCa3.1-tranfected cells, 1-EBIO effect was reduced by 78.94% vs. control (*p* = 9.4 · 10^−13^, Figure 5A). We then investigated the involvement of TRPC1 channels in the Ca^2+^ entry induced by 1-EBIO-mediated KCa3.1 activation. Knockdown of TRPC1 showed a strong reduction in the magnitude of 1-EBIO response (75.97%, *p* = 9.66 · 10^−9^, Figure 5A). Again, the simultaneous knockdown of TRPC1 and KCa3.1 did not result in a significant additive effect (83.96% decrease vs. Ctrl with *p* = 3.82 · 10^−8^, *p* = 0.6 vs. siTRPC1 and *p* = 0.74 vs. siKCa3.1). Taken together, our results suggest that the hyperpolarization-evoked Ca^2+^ entry, due to KCa3.1 activation, is mediated by the TRPC1 channels, and hence supporting our hypothesis of the functional coupling between KCa3.1 and TRPC1.

### TRPC1 and KCa3.1 appear co-localized in lipid rafts

**Figure 6:**
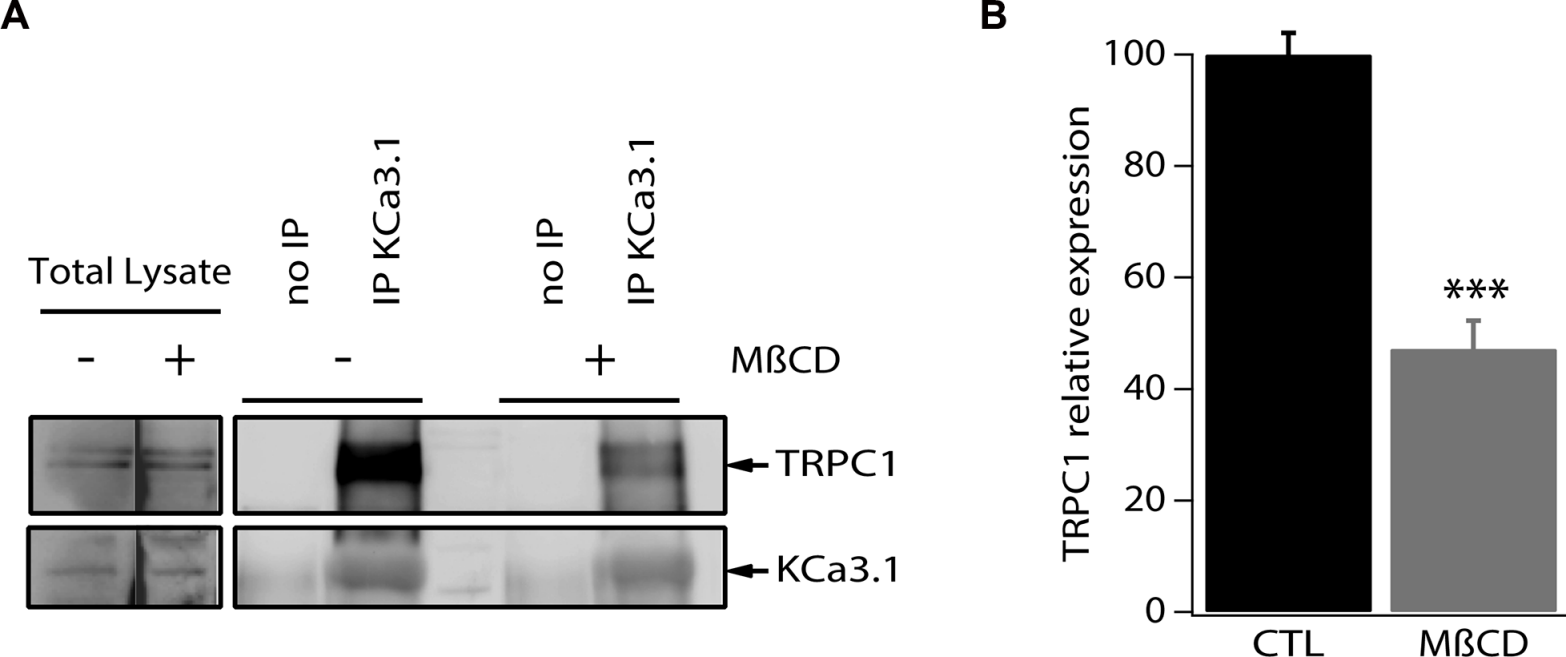
TRPC1 and KCa3.1 appear co-localized in lipid rafts in MCF-7 cells (**A**) MCF-7 cell lysates were subjected to immunoprecipitation with anti-KCa3.1. KCa3.1 and TRPC1 were identified by Western blot analysis using anti-KCa3.1 and anti-TRPC1 antibodies. Cells were treated or not treated with methyl-β-cyclodextrin. (**B**) Quantification of the KCa3.1 and TRPC1 interaction in MCF-7 cells treated or not treated with methyl-β-cyclodextrin. ***p < 0.001.

To test the hypothesis of whether the observed functional coupling is due to protein-protein interaction between TRPC1 and KCa3.1, we conducted immunoprecipitation experiments using a specific KCa3.1 antibody. Total MCF-7 protein lysate was utilized to immunoprecipitate KCa3.1, after which the precipitate was used for immediate western blot and probed with antibodies directed against TRPC1 and KCa3.1. As shown in Figure 6, KCa3.1 precipitate exhibited a pull-down of TRPC1 suggesting a co-localization and interaction between the two channels (Figure 6A).

Various ion channels localize to cholesterol and sphingolipid-enriched regions of the plasma membrane known as lipid microdomains or lipid rafts and increasing evidence suggests that membrane rafts regulate channel function in a number of different ways, including selectively recruiting interacting signalling molecules to generate subcellular compartments that may be important for efficient and selective signal transduction [25–27]. We then set out to examine the co-localization of KCa3.1 and TRPC1 channels in the plasma membrane microdomains. For this purpose, we experimentally disrupted the lipid rafts by pretreatment of MCF-7 cells with methyl-β-cyclodextrin (MβCD), the most commonly used cyclodextrin for cholesterol depletion and hence lipid raft disruption [28–30]. MCF-7 cells were treated with 5 mg/ml MβCD for 24 h at 37°C prior to collecting the cell lysates and performing immunoprecipitation. We found that the treatment with MβCD resulted in a 52.8% decrease of the pulled-down amount of TRPC1 (Figure 6A, 6B). Taken together, our results demonstrate that TRPC1 and KCa3.1 are at least partially co-localized in lipid rafts where they interact and cooperate in regulating Ca^2+^ entry in MCF-7 cells.

### High KCa3.1 mRNA expression correlates to unfavorable clinical parameters and poor patient prognosis in breast cancer

The previous results clearly show the importance of KCa3.1 and TRPC1 function for MCF-7 breast cancer cell proliferation. Both KCa3.1 [11, 13, 21] and TRPC1 [12, 13] have separately been described as involved in breast cancer cell proliferation. In addition, both KCa3.1 and TRPC1 mRNA and protein levels were shown to be higher in breast cancer tissue than in matched normal breast tissue [3, 10]. However, to our knowledge no data have been published on a possible prognostic function for these two genes. Also, no public knowledge exists on a role in the various molecular subtypes for breast cancer, or on the molecular mechanisms by which KCa3.1 and TRPC1 could be involved in breast cancer progression.

**Figure 7:**
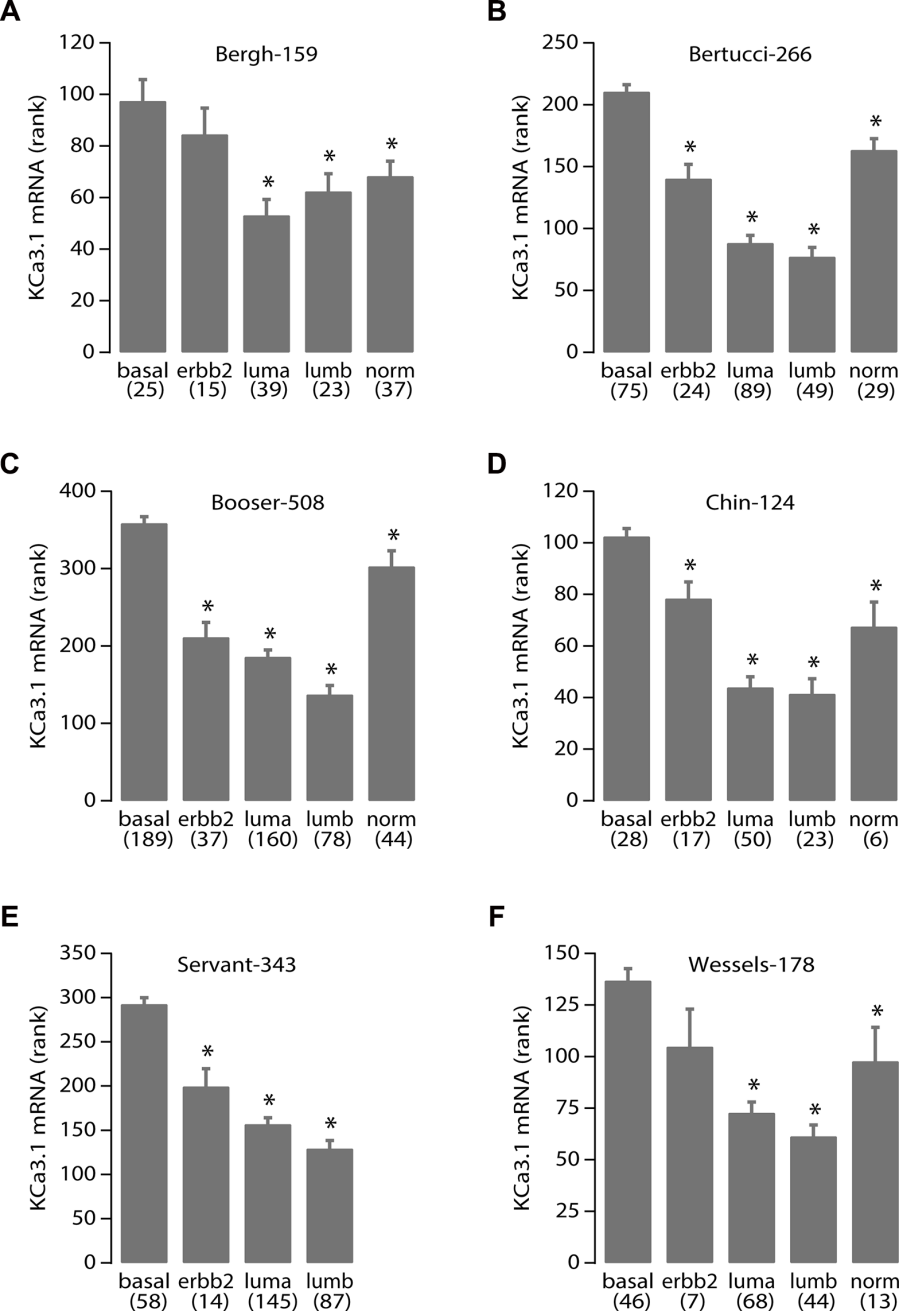
High KCa3.1 expression correlates with the basal breast cancer subtype KCa3.1 tumor mRNA expression correlation with breast cancer molecular subtypes. Panels A-F represent the results from all 6 breast cancer datasets in the public domain that contain data on breast molecular subtypes: Bergh-159 (GSE1456), Bertucci-266 (GSE21653), Booser-508 (GSE25066), Chin-124 (E-TABM-158), Servant-343 (GSE30682), and Wessels-178 (GSE34138), respectively. Below the graph are the different subtypes: basal-like (basal), Her2/Neu/ERBB2 over-expressing (erbb2), luminal-a (luma), luminal-b (lumb) and normal-like (norm) according to [31], between brackets are the number of samples per subtype. Vertical bars represent the S.E.M. The Servant-343 set did not contain samples of the “normal” subtype. More data on the sets are in Table 1 and its legend. Statistical analysis was performed using the non-parametric Kruskal-Wallis test. *denotes significant differences with the basal subtype expression (all *p* < 0.005).

We therefore decided to perform data mining on genome-wide mRNA expression profiles of breast cancer sample sets in the public domain. We were able to download and analyze 17 breast cancer datasets on three different array platforms (Table 1). No clear pattern for TRPC1 mRNA expression emerged. Instead, it seemed to be widely expressed in all breast cancer sets and subtypes (data not shown). However, KCa3.1 showed very distinctive behavior. In 12 of 13 datasets that were annotated for SBR/Elston-Ellis tumor grade, KCa3.1 showed a significantly higher expression in the highest tumor grade (grade 3). Also, it was significantly better expressed in ER-negative than in ER-positive samples (in 15 of 16 annotated datasets), and in PR-negative versus PR-positive samples (in 10 of 11 annotated datasets). Conflicting correlation patterns were not observed in any of the datasets. In sharp contrast, KCa3.1 expression never showed a correlation to HER2/Neu/ERBB2 over-expression. When KCa3.1 mRNA expression was examined between the different molecular breast cancer subtypes [31], it was significantly highest expressed in the aggressive basal-like subtype in all 6 datasets annotated for this parameter (Figure 7) [31–33]. In addition, five datasets contained information on the mutation status of the P53 tumor suppressor gene. In all 5, KCa3.1 mRNA expression was significantly higher in tumors with, than in tumors without P53 gene mutations. The results clearly show that KCa3.1 mRNA expression is higher in tumors with unfavorable (ER-negative, PR-negative, P53-mutated, basal-like) than in tumors with favorable (ER-positive, PR-positive, P53 wildtype, luminal/normal-like) clinical parameters. The expression patterns were consistent over almost all 17 datasets, which had very different tumor subtype and grade compositions, contained patients from diverse geographical regions, and were analyzed on four separate array platforms. We therefore propose that these results are very robust. The correlation of KCa3.1 expression and P53 mutant status in addition offers a possible mechanism for increased proliferation in cells with high KCa3.1 activity.

**Table 1:** KCa3.1 expression correlations in public breast cancer datasets

Dataset		KCa3.1	Grade	ER status	ERBB2 status	PR status	P53 status	Subtype	Platform	GSE	PMID
Name	Size	mRNA		*P* value	*P* value	*P* value	*P* value				
Bergh	159	113.9 (66)	Highest in 3^b^					Highest in Basal^e^	Affymetrix U133A	1456	16280042
Bertucci	266	170.4 (217)	Highest in 3	3.2 · 10^−19^	Not significant	2.0 · 10^−16^	2.2 · 10^−4^	Highest in Basal	Affymetrix U133P2	21653	20490655
Black	107	157.3 (81)	Highest in 3	4.0 · 10^−6^		8.5 · 10^−7^			Affymetrix U133P2	36771	22564725
Booser	508	182.8 (180)	Highest in 3	5.2 · 10^−25^	Not significant	1.9 · 10^−13^		Highest in Basal	Affymetrix U133A	25066	21558518
Chin	124	121.4 (64)	Highest in 3^c^	1.7 · 10^−9^	Not significant	1.1 · 10^−5^	1.1 · 10^−3^	Highest in Basal	Affymetrix U133A	^g^	17157792
Clynes	121	201.1 (35)	Highest in 3^c^	5.7 · 10^−4^					Affymetrix U133P2	42568	23740839
EXPO	351	125.9 (188)	Highest in 3^b^	5.0 · 10^−8^	Not significant	8.7 · 10^−3^			Affymetrix U133P2	2109	^i^
Halfwerk	947	n.d.^a^	Highest in 3	1.3 · 10^−19^	Not significant	7.3 · 10^−14^	1.4 · 10^−10^		Affymetrix U133A	^h^	^i^
Iglehart	123	196.4 (103)	Highest in 3	4.7 · 10^−10^	Not significant				Affymetrix U133P2	5460	18297396
Jonsdottir	94	198.3 (54)	Highest in 3	4.6 · 10^−6^	Not significant				Illumina hwg6v3	46563	24599057
Miller	251	120.4 (108)	Highest in 3	3.1 · 10^−5^		1.9 · 10^−4^	1.4 · 10^−9^		Affymetrix U133A	3494	16141321
Prat	156	144.7 (76)		Trend only^d^	Not significant	Trend only^d^			Affymetrix U133P2	50948	24443618
Servant	343	204.3 (271)	Highest in 3	3.3 · 10^−21^	Not significant	3.3 · 10^−6^	6.7 · 10^−5^	Highest in Basal^f^	Illumina hwg6v3	30682	22271875
Sotiriou	198	94.4 (112)	Trend only	1.7 · 10^−4^					Affymetrix U133A	7390	17545524
TCGA	528	n.d.^a^		4.6 · 10^−34^	Not significant	3.4 · 10^−20^			Agilent 244 K	^j^	23000897
Wang	286	138.6 (169)		2.2 · 10^−17^					Affymetrix U133A	2034	15721472
Wessels	178	115.6 (178)		1.9 · 10^−11^		0.02		Highest in Basal^e^	Illumina hwg6v3	34138	23203637

All 26 breast cancer mRNA expression profiling datasets in the public domain were scrutinized for KCa3.1 (KCNN4 gene) mRNA expression and clinical parameters. 17 datasets were subsequently analyzed using R2. Five datasets with a sample size < 80 (Concha-66 (GSE29431), Desmedt-55 (GSE16391), Loi-77 (GSE9195), Quiles-61 (GSE28844), and Wessels-60 (GSE41656)) and four datasets with missing clinical parameters (Bos-204 (GSE12276), Miller-116 (GSE5462), Sotiriou-120 (GSE16446), and Zhang-136 (GSE12093), were omitted from analysis Data were downloaded and analyzed as described in the Materials and Methods. The first two columns represent name and size of the dataset. KCNN4 describes the average present KCa3.1 mRNA expression values in the datasets, with between brackets the amount of samples in the dataset that show significant expression. Grade represents the KCa3.1 expression in the three grades (1–3) according to Elston-Ellis/SBR [63]. ER, ERBB2 (Her2/Neu), and PR status describe the KCa3.1 expression in receptor-negative versus receptor-positive samples; the *P* values denote significantly higher expression in receptor negative samples. P53 status describes the KCa3.1 expression in tumours with versus tumours without P53 gene aberrations; positive *P* values denote significantly higher expression in tumours with P53 aberrations. Subtypes describe KCa3.1 expression in the five breast cancer molecular subgroups (basal-like, ERBB2-overexpressing, luminal-a, luminal-b, and normal-like) according to [31]. Platform lists the kind of arrays used, GSE shows the NCBI GEO set number, and PMID the PubMed ID of the array publication.

a Dataset expression values do not allow distinction between present and absent expression.

b Expression is significantly higher in grade 3 versus grade 2, and in grade 2 versus grade 1, but only trends in grade 3 versus grade 1.

c Expression is significantly higher in grade 3 versus grade 2, but only trends in the other comparisons.

d In this dataset, the amounts of receptor-negative and receptor-positive samples are the opposite of the normal distribution, which could cause the non-significant comparison result.

e The difference between basal-like and ERBB2-overexpressing trends, but is not significant.

f This dataset does not contain “normal-like” samples.

g E-TABM-158.

h This dataset compiles the Chin-123 (also analyzed separately), Desmedt-147 (GSE7390), Loi-178 (GSE6532), Miller-247 (GSE3494), Minn-96 (GSE5327), and Pawitan-156 (GSE1456) datasets.

i This dataset has not yet been published. All comparisons were made using the Kruskal-Wallis test.

j TCGA: http://tcga.cancer.gov/dataportal.

**Figure 8:**
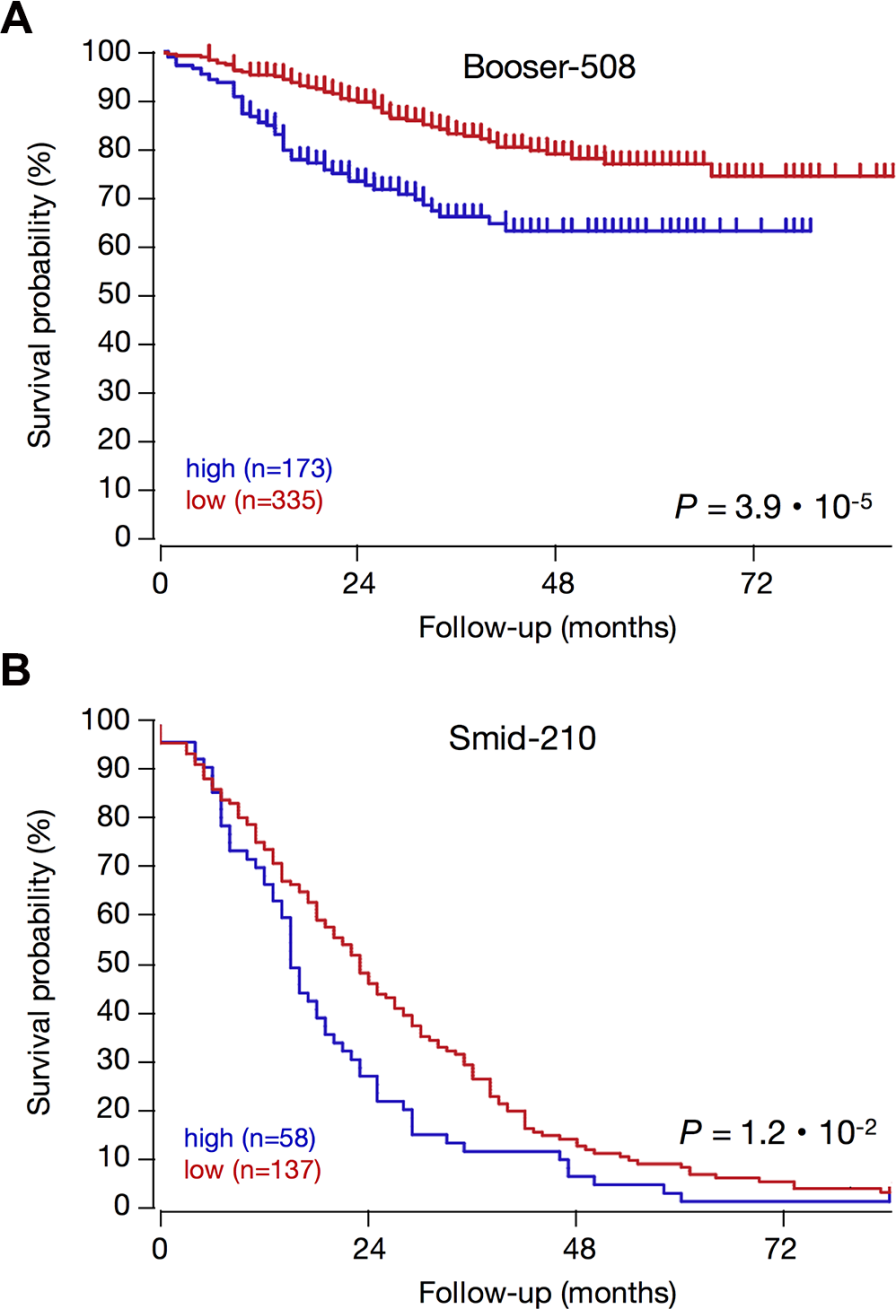
High KCa3.1 expression is prognostic for poor patient outcome in breast cancer KCa3.1 expression correlation with survival: Kaplan–Meier graphs representing the survival prognosis of breast cancer patients based on high or low KCa3.1 tumor mRNA expression. (**A**) Kaplan-Meier analysis in the Booser-508 primary breast cancer dataset (censored at 84 months): upon dividing the patient group on average expression, those with high KCa3.1 tumor mRNA expression (*n* = 173) had a survival probability of 63%, while those with low KCa3.1 expression (*n* = 335) had a significantly higher survival probability of 75% (*p* = 3.9 · 10^−5^). Also when the patient group was divided on median KCa3.1 expression, the difference was significant (*P* = 7.6 ·10^−4^, not shown). (**B**) Kaplan-Meier analysis in the Smid-210 dataset of patients with known relapse (not censored): division of the patient group on average expression showed that the survival period of patients with low KCa3.1 tumor mRNA expression (*n* = 137) is significantly longer than that of patients with high KCa3.1 expression (*n* = 58) (*p* = 9.5 · 10^−3^). Graphs are drawn up to 84 months. Patients at risk data during the analysis interval are not available for these datasets on the R2 website. The two other breast cancer datasets in the public domain that contain survival data (Bergh-159; GSE1456, and Bertucci-266; GSE21653) showed significant correlations between high KCa3.1 tumor mRNA expression and poor outcome in several different groupings, but these lost significance after Bonferoni correction for multiple testing (not shown).

The ER/PR, P53, and subtype status of tumors with high KCa3.1 expression suggest that KCa3.1 is correlated to rapid tumor growth and spread, and poor patient outcome. To establish the prognostic value for KCa3.1 tumor expression, we analyzed all four public breast cancer datasets that had survival data. Indeed, in Kaplan-Meier graphs, high KCa3.1 tumor expression was significantly prognostic for poor outcome in two datasets (Figure 8). Interestingly, KCa3.1 expression was prognostic in patients that had a long average survival (in the Booser-508 dataset, panel A), as well as in primary tumors of patients that were known to later relapse and therefore had only short average survival (in the Smid-210 dataset, panel B). In the two other datasets with survival data, Bergh-159 and Bertucci-266, the correlation between high KCa3.1 tumor mRNA expression and poor outcome showed similar trends, but these did not reach statistical significance (not shown). The observation that KCa3.1 is a prognostic marker in breast cancer is therefore preliminary, and awaits the analysis of more public data when they become available.

Together, these data strongly suggest that KCa3.1 has an important role in the breast cancer cell, in particular in tumors that are not amenable to standard (hormone) treatment, and that have accumulated DNA repair defects. This strengthens the case for KCa3.1 as a potential target for novel drug development and warrants further investigation in different breast cancer subtypes.

## DISCUSSION

The major findings of this study are 1. KCa3.1 channel is a key regulator of breast cancer cell cycle progression and proliferation and its high mRNA expression in breast cancer patients is associated with unfavorable clinical parameters and poor prognosis, which makes this channel an attractive target for developing new therapy, and 2. KCa3.1 regulates Ca^2+^ influx and cell proliferation by functional cooperation with TRPC1 channel in our MCF-7 cell model. The expression of both channels increases at the end of G1 phase and silencing either KCa3.1 or TRPC1 reduces cell proliferation, accumulates cells in G1 phase, and reduces both basal Ca^2+^ entry and Ca^2+^ entry activated by 1-EBIO (KCa3.1 activator). Additionally, co-immunoprecipitation experiments showed that TRPC1 and KCa3.1 are, at least partially, colocalized in the plasma membrane, likely in the lipid rafts where they may cooperate to regulate Ca^2+^ entry and thus G1 phase progression and cell proliferation.

KCa3.1 channel expression has been shown to be upregulated in many cancers, where it promoted cell growth and cell cycle progression [11, 13, 34, 35]. Several studies have shown that the activity of KCa3.1 channels facilitates the Ca^2+^ influx by hyperpolarizing the cell membrane and increasing the driving force for Ca^2+^ influx in different cell types [36–38]. In breast cancer MCF-7 cells, our previous work has demonstrated that when cells progress through G1 phase, they show an increase of KCa3.1 expression level and current density paralleled by a highly negative resting membrane potential (RMP) and a higher basal Ca^2+^ concentration [Ca^2+]^_i_ [14]. Pharmacological inhibition of KCa3.1 channels reduces the concentration of intracellular Ca^2+^ and depolarizes the RMP in cells accumulated at the end of G1 phase [39]. We have also showed that the decrease of the extracellular Ca^2+^ concentration [Ca^2+^]_o_ inhibits MCF-7 cell proliferation suggesting that Ca^2+^ influx is essential for BC MCF-7 cell proliferation [40]. TRPC1 is an important Ca^2+^ regulator in a variety of cell types including malignant gliomas [41], ovarian cancer [42], and lung cancer [43, 44]. In the MCF-7 breast cancer cell line, we previously demonstrated that TRPC1 provides a Ca^2+^ entry that regulates cell proliferation via ERK1/2 phosphorylation [16]. Here, we show that silencing of TRPC1 induced G0/G1 cell cycle arrest resulting in a significant decrease of cell proliferation. The fact that TRPC1 and KCa3.1 knockdown similarly affected cell proliferation and cell cycle distribution strongly favored the hypothesis that TRPC1 channels may be the key actors in Ca^2+^ influx induced by KCa3.1 activation. This was confirmed by Mn^2+^ quenching, where silencing either TRPC1 or KCa3.1 markedly decreased Ca^2+^ entry in our culture condition. Moreover, when TRPC1 was downregulated, the amount of Ca^2+^ entry in response to the KCa3.1 activator (1-EBIO) has significantly decreased suggesting that TRPC1 channels contribute to the Ca^2+^ entry induced by KCa3.1 channels activation (thus RMP hyperpolarization).

KCa3.1 channels control G1 (mainly late G1) phase, G1/S transition, and G2/M phase in several types of cancer including breast, prostate, endometrial and colon [12, 13, 35, 45]. In breast cancer, the activation of KCa3.1 contributes to the progression of cell through G1, and to G1/S transition [39]. TRPC1 has been also reported to play an essential role in cell cycle progression especially in G1 phase [44, 46]. Indeed, an upregulation of TRPC1 protein level in the G1 compared with G0 was observed in Ehrlich Lettré Ascites (ELA) cells [46] while TRPC1silencing in non-small cell lung carcinoma cell lines decreased cyclin D1 and D3 expression levels, resulting in G0/G1 cell cycle arrest [44]. Here, we show that both TRPC1 and KCa3.1 expression is increased at the end of G1 (Figure 3). Our results highlight a common pathway or a signaling connection between KCa3.1 and TRPC1 in the regulation of G1/S transition.

Several studies have reported a physical interaction between Ca^2+^-activated K^+^ (KCa) channels with several Ca^2+^ partners including TRP channels [12, 47–50]. For example, in vascular smooth muscle cells, TRPC1 is physically associated with BK_Ca_ and Ca^2+^ influx through TRPC1 activates BK_Ca_ to induce membrane hyperpolarization [49]. A similar interaction was proposed between KCa3.1 channels and store-operated calcium channels in macrophages. In this way, a small Ca^2+^ influx current induces a large KCa3.1 current providing the necessary electrochemical driving force for prolonged Ca^2+^ signaling and store repletion [51]. Furthermore, it has been shown that the TRPC1-mediated Ca^2+^ entry is the primary regulator of KCa3.1 channel in human salivary gland cells as it induces KCa3.1 channel activation leading to NFkB signaling and cell proliferation [52]. The TRPC1/KCa3.1 connection has been reported as an important Ca^2+^ signaling mechanism in the regulation of cancer cell migration [53, 54], but so far only one study has reported a link between KCa3.1 and TRP channels in cancer cell proliferation. Indeed, in the prostate cancer cell line LNCaP, TRPV6 has been proposed as a major source of passive Ca^2+^ influx in response to the hyperpolarization associated with KCa3.1 channels activation [12]. No specific interaction inhibitors exist that would allow to study the relationships between the physical and functional interactions of KCa and TRP channel proteins by disturbing the physical interactions. However, in the present study, we did find that KCa3.1 physically associates with TRPC1, and is at least partially co-localized in the lipid rafts, suggesting that this close proximity allows TRPC1 channels to control the KCa3.1 activation-mediated Ca^2+^ entry at least in part. We expressly note that in addition to the KCa3.1-TRPC1 interaction, other Ca^2+^-regulating proteins are known to co-assemble with TRPC1, and might be involved in the Ca^2+^ signaling. These include other TRP channels, but also IP3, STIM1, and ORAI1. [22, 55, 56].

The results described above clearly show that the KCa3.1 expression and the KCa3.1/TRPC1 interaction are involved in proliferation of the MCF-7 breast cancer cell. In an effort to translate cell line experiment data to an *in vivo* patient situation, we analyzed KCa3.1 and TRPC1 mRNA expression in public breast cancer patient sample sets. KCa3.1 and TRPC1 mRNA and protein levels have previously been shown to be higher in breast cancer than in matched normal tissue, but this was a single patient series without information on clinical and pathological breast cancer parameters or patient survival [3, 10]. Our mining of 17 public breast cancer datasets showed that KCa3.1 tumor mRNA expression is significantly correlated to unfavorable breast cancer parameters: negative ER and PR status, P53 gene aberration, and the aggressive basal-like molecular subtype. Indeed, a preliminary analysis for prognostic value of KCA3.1 tumor mRNA expression showed that in two of the four separate survival analyses available in the public domain, high KCa3.1 tumor mRNA expression was significantly prognostic for poor survival. These consistent results represent the most extensive KCa3.1 expression analysis in breast cancer so far.

While the molecular mechanisms regulating KCa3.1 and hormone receptor expression in breast cancer cells are yet unclear, the significant correlation between p53 mutant status and high KCa3.1 expression we found fits well in the knowledge on KCa3.1 function and cell cycle. In MCF-7 cells, high KCa3.1 expression is found in (late) G1 and is essential for G1 to S transition. In contrast, KCa3.1 (or TRPC1) knockdown resulted in a G1 block. This block is most probably caused by P53, as KCa3.1 knockdown has been shown to cause P53 activation, resulting in e.g. P21 accumulation in prostate cancer cells [12], and MCF-7 cells have wildtype P53 [57]. In aggressive breast cancer, the absence of functional P53 would untether the effect of KCa3.1 on the cell cycle.

The high KCa3.1 expression in especially those breast cancer subtypes that cannot be treated with existing hormone therapy and/or have poor outcome warrants further investigation. Testing of known or novel KCa3.1 inhibitors, possibly in combination with classical DNA-targeting drugs, might well unveil an essential role for KCa3.1 expression in breast cancer growth and survival, and yield a venue of treatment yet unavailable for patients suffering from aggressive, basal-like or triple-negative breast cancer.

## MATERIALS AND METHODS

### Cell culture and cell synchronization

The MCF-7 breast cancer cell line was obtained from the American Type Culture Collection (LGC Standards, Molsheim, France). MCF-7 cells were grown in Eagle's Minimum Essential Medium (EMEM; Gibco, Life Sciences, Cergy Pontoise, France) supplemented with 5% fetal bovine serum (FBS), 2 mM L-glutamine, and 50 μg/ml gentamicin (all from Gibco). The culture medium was changed every 2 days. Cells were maintained at 37°C in a humidified atmosphere with 5% CO_2_.

To obtain synchronized cell cultures accumulated in the early or late G1 phase, MCF-7 cells were cultured for 24 h with serum- and phenol red-free EMEM [39, 58], or with complete EMEM medium supplemented with 2 mM thymidine [39], respectively.

### Cell transfection and RNA interference

Transfection of cells was performed using nucleofection technology (Amaxa Biosystems, Lonza, Aubergenville, France) according to the protocol as previously described [40]. Cells were transiently transfected with siRNA directed against TRPC1 and KCa3.1 [13, 16], or with scrambled siRNA as a control (siCTL) (siGENOME Non-Targeting siRNA, Dharmacon Research, Chicago, IL), and used 72 h after transfection.

### Cell proliferation assay

MCF-7 cells were grown in 35 mm Petri dishes in complete EMEM medium. The involvement of TRPC1 and KCa3.1 in cell proliferation was investigated on MCF-7 cells transfected with siRNA as described above. Cell proliferation was determined by counting cells using a Trypan Blue assay. Mortality was scored by simultaneous counting of darkly stained cells (and never reached > 5% overall). The results were expressed as the percentage of proliferating cells in the experiment normalized to the control condition.

### Cell cycle analysis

For the measurement of the cellular DNA content, cells transfected by siTRPC1 and siKCa3.1 or siCTL were pelleted, re-suspended in PBS/EDTA, treated with 20 μg/ml of RNase A (Sigma-Aldrich, Lyon, France) and stained with 50 μg/ml of propidium iodide (Sigma-Aldrich). Samples were then analyzed using an Elite flow cytometer (Beckman Coulter, Villepinte, France). The percentage of cells in different phases was calculated using WinMDI 2.8 (Purdue University, West Lafayette, Indiana) and Cylchred (Cardiff University, Cardiff, UK) software.

### qRT-PCR

Quantitative reverse transcriptase PCR (qRT-PCR) was performed as previously described [16]. Sense and anti-sense PCR primers specific to KCa3.1 (sense: GCAGGAACTGGCATTGGACT, anti-sense: CTGATCGTGCATTTAACCAGGA), TRPC1 (sense: GAGGTGATGGCGCTGAAGG, anti-sense: GCACGCC AGCAAGAAAAGC) and β-actin (sense: CAGAGCAA GAGAGGCATCCT, anti-sense: ACGTACATGGCTGGG GTG) were used. TRPC1 and KCa3.1 mRNA levels in breast cancer cells were normalized to the endogenous control (β-actin) using the Pfaffl method [59]:

$$\text{Ratio}=\frac{{\left({\text{E}}_{\text{target}}\right)}^{\Delta \text{Ct target }\left(\text{siCTL}-\text{siTarget}\right)}}{{\left({\text{E}}_{\text{ref}}\right)}^{\Delta \text{Ct ref }\left(\text{siCTL}-\text{siTarget}\right)}}$$

### Calcium imaging

After transfection, cells were directly seeded on glass coverslips, and grown for an additional 72 h. Cells were then incubated in culture medium containing 3 μM Fura-2 AM (Sigma Aldrich) for 45 minutes at 37°C before Ca^2+^ measurement. After Fura loading, cells were washed and kept in extracellular saline solution containing 145 mM NaCl, 5 mM KCl, 2 mM CaCl_2_, 1 mM MgCl_2_, 5 mM glucose 5, 10 mM HEPES-NaOH, at pH 7.4. The glass coverslip was mounted in a chamber on a Zeiss (Marly-le-Roi, France) microscope equipped for fluorescence. Fura-2 fluorescence was excited at 340 and 380 nm using a monochromator (Polychrome IV; TILL Photonics, Planegg, Germany), and captured by a Cool SNAPHQ camera (Princeton Instruments, Evry, France) after filtration through a long-pass filter (510 nm). Metafluor software (version 7.1.7.0, Molecular Devices, St. Grégoire, France) was used for acquisition and analysis. The [Ca^2+^]_I_ concentration was derived from the ratio of the fluorescence intensities for each of the excitations wavelengths (F_340_/F_380_). All recording were made at room temperature. The cells were continuously perfused with the saline solution. The flow rate of the whole cell chamber perfusion system was set to 1ml/min and the chamber volume was 700 μl.

### Manganese influx measurements

MCF-7 cells were plated on glass coverslips after transfection with siRNA-Control (siCTL) or siTRPC1 and siKCa3.1. To estimate divalent influx through the plasma membrane, we used the manganese-induced quenching of Fura2 approach as previously described [40]. Briefly, cells were loaded with 3 μM Fura-2 AM in the growth medium at 37°C for 45 min. After a period of 1 minute, the Ca^2+^ (2 mM) present in the perfusion medium was replaced by 2 mM Mn^2+^. To measure Mn^2+^ influx, cells were excited at 360 nm and the emission signal was recorded at 510 nm at one second intervals. The Mn^2+^ influx was estimated from the quenching rate of 360 nm fluorescence.

### Co-immunoprecipation

MCF-7 cells were lysed in RIPA buffer (1% Triton X-100, 1% sodium deoxycholate, 150 mM NaCl, 50 mM Tris-HCl pH 7.4, 2 mM EDTA, 0.5 mM sodium orthovanadate, and P8340 inhibitor cocktail (Sigma-Aldrich)). 1 mg of MCF-7 protein lysates were precleared for 30 min with proteins A and G sepharose magnetic beads and then incubated over night with 3 μg of anti-KCa3.1 antibody (sc-32949, Santa Cruz, Santa Cruz, CA). The antigen-antibody complexes were precipitated with proteins A and G sepharose magnetic beads (Millipore, PureProteome™ Magnetic Beads) for one hour. After denaturation, proteins were separated by denaturing SDS–PAGE and transferred onto nitrocellulose membranes. KCa3.1 was detected using anti-KCa3.1 antibody (sc-32949, Santa Cruz, at 1:500) and anti-rabbit secondary antibody (TrueBlot Anti-Rabbit IgG HRP 18-8816, eBioscience, Paris, France, at 1:1000). Anti-TRPC1 anti-body (ACC-010, Alomone, Jeruzalem, Israel, at 1:200) was used for TRPC1 detection. Bands were detected using an enhanced chemiluminescence kit (GE Healthcare, Saclay, France) and quantified using the densitometric analysis option in the Bio-Rad image acquisition system (Bio-Rad Laboratories, Marnes-la-Coquette, France). For lipid rafts disruption induced by surface cholesterol depletion, MCF-7 cells were treated with 5 mg/mL of Methyl-β-cyclodextrin (Sigma-Aldrich) for 24 h in complete culture medium.

### Public breast cancer dataset analyses

Human breast cancer genome-wide expression profile datasets from patient sample series deposited for public access in a MIAME-compliant format were obtained through the Gene Expression Omnibus (GEO) database at the NCBI website [60], except for the Chin-124 dataset (E-TABM-158), which was from EMBL-EBI ArrayExpress (www.ebi.ac.uk/arrayexpress). Full data on the profiling experiments and the patient cohort clinical data for all datasets are available at the GEO/EMBL-EBI sites through their GSE/E-TABM and PubMed PMID numbers, listed in Table 1 and its legend. CEL data were analyzed as described in [61]. Briefly, gene transcript levels from studies using Affymetrix arrays were determined from data image files using GeneChip operating software (MAS5.0 and GCOS1.0, from Affymetrix). Samples were scaled by setting the average intensity of the middle 96% of all probe-set signals to a fixed value of 100 for every sample in the dataset, allowing comparisons between micro-arrays. The Servant-343 (GSE30682), Jonsdottir-94 (GSE46563), and Wessels-178 (GSE34138) studies used Illumina arrays, and underwent custom processing and normalization as described on their GSE pages at the GEO site. The TCGA-528 study used a custom Agilent 244 K array, as described on http://tcga.cancer.gov/dataportal. The TranscriptView genomic analysis and visualization tool within R2 was used to verify that probe-sets had an anti-sense position in an exon of the gene (http://r2.amc.nl > genome browser). The Affymetrix probe-sets selected for KCA3.1 (KCNN4 gene; 204401_at for Affymetrix arrays and ILMN_1709937 for Illumina arrays), and for TRPC1 (205802_at/205803_s_at and ILMN_1674380, respectively) meet these criteria and showed significant expression in > 25% of samples in each dataset. Analyses were performed using R2; a genomics analysis and visualization platform developed by Jan Koster in the Department of Oncogenomics at the Academic Medical Center – University of Amsterdam (http://r2.amc.nl).

### Statistical analysis

Data are presented as means ± SE (*n* = number of individual measurements). Plots were produced using Igor Pro (by WaveMetrics, Lake Oswego, OR Inc.). Statistical analysis of the data was performed using ANOVA (Figures 1, 2 4 and 5) or paired *t*-tests (Figures 3 and 6) as appropriate. KCa3.1 mRNA expression correlation with breast cancer clinical and genetic features (Figure 7 and Table 1) were determined using the non-parametric Kruskal-Wallis test, to prevent undue influence of non-normally distributed data. KCa3.1 mRNA expression correlation with survival probability (Figure 8) was evaluated by Kaplan-Meier analysis using the log-rank test as described [62]. For all tests, differences were considered significant if *p* < 0.05.
